# Mechanistic Investigation of Microdroplet Formation in High-Viscosity Shear-Thinning Hydrogel Bioinks

**DOI:** 10.3390/gels12020148

**Published:** 2026-02-06

**Authors:** Qiang Gao, Yanling Mi, Kaicheng Yu, Youyun Shang, Lihua Lu, Yongqiang Gao, Peng Zhang

**Affiliations:** School of Mechatronics Engineering, Harbin Institute of Technology, Harbin 150001, China; gaoq@hit.edu.cn (Q.G.);

**Keywords:** hydrogel bioink, 3D printing, shear-thinning behavior, inkjet-based dispenser

## Abstract

High-resolution biofabrication requires precise microscale deposition, yet drop-on-demand (DOD) inkjet bioprinting is constrained by a narrow printable viscosity window. Many biocompatible hydrogel precursors display high zero-shear viscosity and strong shear-thinning, so stable droplet ejection typically requires dilution or reformulation that can compromise the biochemical microenvironment. We present a transient shear-enabled jetting method that exploits intrinsic shear-thinning by using a high-frequency electromagnetic microvalve to deliver short, high-pressure pulses. The resulting localized shear dynamically lowers apparent viscosity in the nozzle region and promotes controlled nucleation, ligament formation, necking, and pinch-off. A coupled, rheology-informed modeling framework (axisymmetric transient CFD, valve dynamics, and electromagnetic FEM) links actuation parameters to droplet volume and stability and guides hardware optimization. Experiments with 2.5% (*w*/*v*) sodium alginate validate stable droplet generation and tunable droplet size via stroke length and driving conditions. These results define a practical process window for high-resolution droplet printing of high-viscosity shear-thinning hydrogel inks.

## 1. Introduction

Organ damage and failure are common complications associated with major diseases such as heart valve disorders and late-stage cancers. Organ transplantation remains the most effective clinical intervention [1,2,3]; however, the severe shortage of donor organs results in many patients losing the opportunity for timely treatment [4,5]. To alleviate this shortage, significant effort has been devoted to the construction of functional artificial tissues and organs [6,7,8]. Among various fabrication strategies, three-dimensional (3D) bioprinting has emerged as a powerful technique capable of generating structurally complex tissue-like architectures with high precision [9].

An ideal bioink should combine high printability, biocompatibility, and mechanical stability while supporting key cellular processes, including adhesion, migration, proliferation, and differentiation [10]. In bioprinting, high-viscosity bioinks markedly improve dimensional accuracy, enable the fabrication of complex architectures, and enhance the mechanical stability of printed constructs [11,12,13]. Their elevated yield stress and storage modulus are critical for preserving structural fidelity and mechanical integrity, particularly in large-scale or load-bearing tissue scaffolds [14]. In addition, high-viscosity bioinks allow printing at high cell densities, thereby providing a microenvironment that more closely resembles in vivo conditions and promotes tissue functionality. The rheological properties of these bioinks can be further optimized through strategies such as composite formulations or multi-network designs to meet the requirements of complex printing processes [15]. When integrated with support-bath printing techniques, high-viscosity bioinks also enable the fabrication of overhanging and high-resolution structures, substantially expanding their application range [16]. Despite these advantages, the use of high-viscosity bioinks places more stringent demands on printing equipment and process parameter control.

Among available bioprinting modalities, drop-on-demand (DOD) inkjet printing offers high spatial resolution, fast deposition, material efficiency, and excellent cell compatibility, making it particularly attractive for microscale tissue construction [17]. However, the jetting performance of DOD systems is strongly dictated by the rheology of the bioink. Printable bioinks typically require a viscosity within 3–30 mPa·s [18], whereas many biocompatible hydrogel precursors (e.g., alginate, gelatin, GelMA) exhibit zero-shear viscosities ranging from several hundred to several thousand mPa·s and show pronounced shear-thinning behavior [19,20]. This large mismatch between intrinsic material rheology and the printable window of DOD inkjetting severely limits the direct use of high-viscosity hydrogel bioinks.

To address this challenge, existing studies primarily pursue two directions: rheological tuning of bioinks and enhancement of jetting actuation. The former includes modifying bioink formulations or processing conditions, such as adjusting temperature [21], adding surfactants [22], or regulating cell concentration [23], to reduce apparent viscosity, but these strategies may alter the biochemical microenvironment or affect cell viability. The latter seeks to extend the printable viscosity range by strengthening the driving mechanism, for example, via electrohydrodynamic jetting [24], microvalve actuation [25], or direct-volume-controlled DOD systems [26]. Although effective to some extent, these methods often require complex and highly customized hardware, limiting system versatility and rapid adaptability to diverse emerging bioinks [27].

For overcoming the fundamental incompatibility between high-viscosity hydrogel precursors and the limited viscosity window of DOD jetting, while avoiding modifications to bioink chemistry, this study proposes a transient jetting strategy that leverages the intrinsic shear-thinning behavior of high-viscosity bioinks. The core idea is to induce strong, localized shear during the short-duration actuation of a high-frequency electromagnetic valve, thereby dynamically reducing the apparent viscosity within the jetting region and enabling stable droplet formation without altering the bioink formulation.

In order to elucidate the coupled “material-flow-actuation” mechanism, we developed a two-dimensional axisymmetric transient CFD model to analyze droplet nucleation, ligament formation, necking, and pinch-off dynamics. A dynamic model of the electromagnetic valve was established to quantify the influence of stroke length, spring preload, and stiffness on the transient motion of the mobile anchor. Finite-element electromagnetic field simulations were further conducted to optimize coil height, ampere-turns, and anchor geometry. Based on these analyses, an electromagnetic-valve-actuated jetting prototype was constructed and integrated into a three-axis Cartesian platform. Experimental validation using a 2.5% sodium alginate solution confirmed the system’s ability to stably jet high-viscosity shear-thinning bioinks. This work establishes a generalizable engineering pathway for inkjet printing of high-viscosity bioinks without altering their biochemical composition, providing a foundation for rapid adaptation and high-precision fabrication of next-generation functional bioinks.

## 2. Results and Discussion

### 2.1. The Shear-Thinning Behavior of High-Viscosity Biomaterials

**Figure 1:**
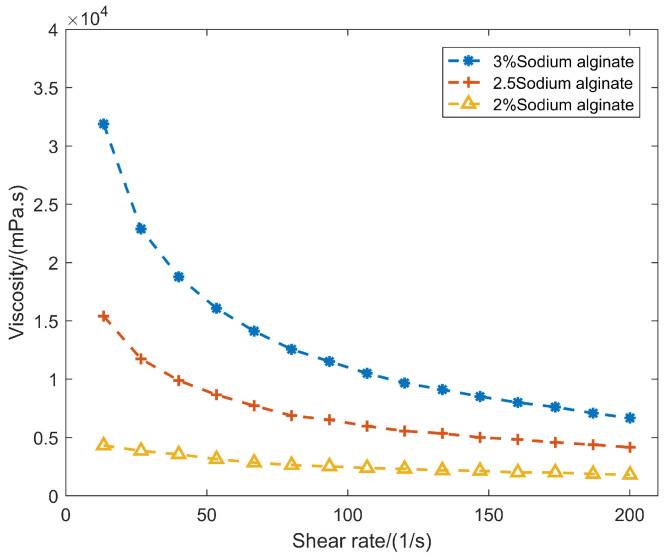
Viscosity curve of sodium alginate at varying concentrations.

The common biomaterials such as sodium alginate are pseudoplastic fluids that exhibit shear-thinning behavior. In this study, a 2.5% sodium alginate solution was adopted as a high-viscosity biomaterial, and its viscosity at different shear rates was measured using a rheometer to characterize its rheological properties. As shown in Figure 1, a power-law relationship between shear rate and viscosity can be obtained. This indicates that the viscosity of the 2.5% sodium alginate solution is not constant; instead, it decreases as the shear rate increases.

Compared with conventional inkjet-based and extrusion-based 3D bioprinting devices, the electromagnetic-valve-actuated dispenser can generate a higher biomaterial velocity driven by the mobile anchor. This produces a higher shear rate in the biomaterial near the nozzle, which in turn reduces the local apparent viscosity. The lower the viscosity around the nozzle, the easier it is to jet the solution successfully, indicating the feasibility of printing high-viscosity biomaterials.

### 2.2. CFD Analysis of the Printing Process

#### 2.2.1. The Formation Mechanism for the Droplets

**Figure 2:**
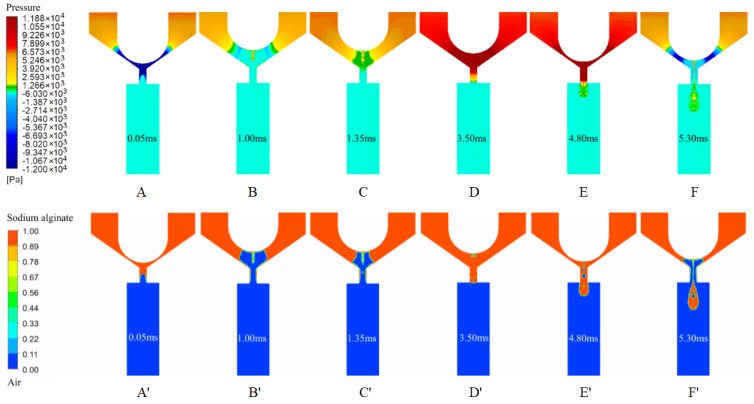
Pressure and liquid–gas phase contours during the printing process. (**A**,**A’**) Valve opening creates negative pressure and a concave meniscus; (**B**,**B’**) Back pressure dominates and the interface bulges; (**C**,**C’**) Flow convergence sustains the jet; microbubbles may be trapped; (**D**,**D’**) Valve closing drives outflow and forms a liquid column; (**E**,**E’**) The column advances and the droplet expands; (**F**,**F’**) Supply cutoff induces necking (Rayleigh–Plateau) and breakup.

During a typical jetting process, droplet formation occurs through a series of well-defined stages. Upon valve opening and initial acceleration, the mobile anchor is driven away from the valve seat by electromagnetic forces. This motion rapidly increases the effective volume of the valve chamber and generates a transient negative pressure. Consequently, a pressure difference develops between the nozzle interior and the surrounding environment, deforming the free liquid surface from an approximately planar configuration into a concave profile (Figure 2A).

The system then transitions to a stage of stable liquid supply accompanied by outward bulging of the free surface. After the anchor reaches its maximum displacement, the negative pressure within the valve chamber gradually dissipates, and back pressure becomes dominant, resulting in a nozzle pressure exceeding the ambient pressure. Liquid from both sides of the flow channel converges toward the center, continuously replenishing the jet (Figure 2B,C). During this stage, a small fraction of air that cannot escape in time becomes entrapped, forming microbubbles.

As the anchor moves back to close the valve, the combined effects of anchor motion and back pressure maintain an elevated internal nozzle pressure, forcing liquid out of the nozzle. This process produces a crescent-shaped free surface followed by the formation of a continuous liquid column (Figure 2D). Driven by inertia and gravity, the ejected liquid advances along the jetting direction, while surface tension promotes radial expansion of the forming droplet (Figure 2E).

When the anchor reverses direction and moves upward, the upstream liquid supply is rapidly interrupted. The flow velocity near the valve seat decreases sharply, whereas the liquid column outside the nozzle continues to move downward due to inertia. This imbalance induces a retraction flow opposite to the jetting direction and generates a localized negative pressure near the nozzle exit. As a result, the outflow is weakened and the rear neck of the liquid column is stretched. The column then enters a surface-tension-dominated necking stage and exhibits the characteristic Rayleigh–Plateau instability, ultimately leading to droplet breakup (Figure 2F).

**Figure 3:**
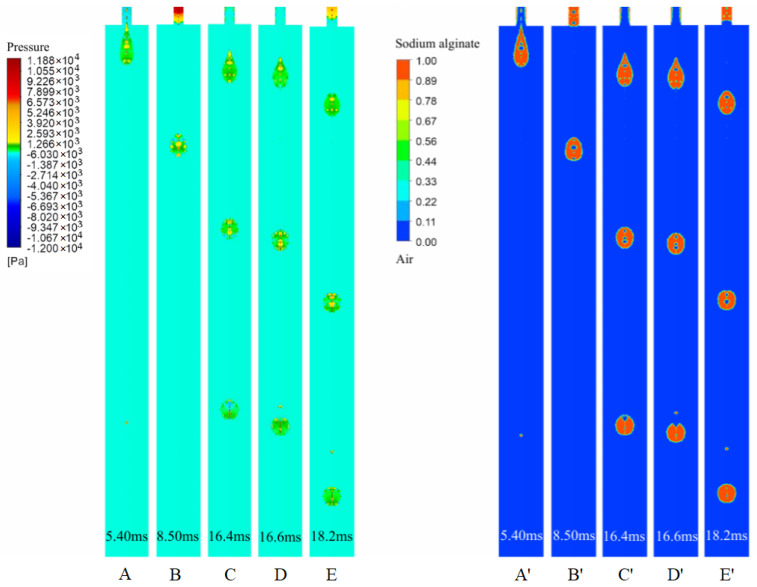
Pressure and liquid–gas phase contours for droplet formation. (**A**,**A’**) Neck thinning leads to rupture; (**B**,**B’**) The primary droplet relaxes toward a spherical shape; (**C**,**C’**) Entrapped bubbles migrate to the interface; (**D**,**D’**) Bubbles escape during flight; (**E**,**E’**) The droplet interface stabilizes.

In the steady liquid-supply and free-surface bulging stage (Figure 3), the transient negative pressure in the valve chamber gradually dissipates once the mobile anchor reaches its maximum displacement and briefly pauses. The back pressure then regains dominance, raising the nozzle pressure above the ambient level. Liquid converges toward the nozzle center from both sides of the channel, continuously replenishing the fluid at the outlet. Because air evacuation lags behind liquid motion, a small amount of air becomes trapped within the fluid, forming microbubbles.

The liquid neck continues to thin until surface tension overcomes inertial and viscous forces, leading to neck rupture (Figure 3A). Once detached from the nozzle, the primary droplet gradually relaxes from an elongated configuration to a nearly spherical or slightly spheroidal shape during its flight (Figure 3B). Interfacial oscillations combined with local inertial effects can generate satellite droplets, some of which subsequently merge with the primary droplet or are reabsorbed by the liquid remaining in the nozzle. During the subsequent flight and gas-release stages, entrapped gas bubbles migrate toward the droplet interface under pressure gradients and eventually escape (Figure 3C,D). Concurrently, satellite droplets may coalesce or break up due to the competing effects of viscous dissipation and surface tension. Ultimately, the primary droplet interface reaches a stable state (Figure 3E), completing the droplet formation, necking, and detachment process.

The transient simulations reveal that the actuation-driven flow generates substantial and dynamically varying shear rates in the nozzle region, which is central to exploiting the shear-thinning behavior for droplet ejection. While the present study focuses on the cell-free jetting process, quantifying the maximum shear stress and its implications for cellular viability (e.g., for fibroblasts or stem cells) is an important aspect for future biological applications. Such characterization will be conducted in subsequent work involving cell-laden bioinks, where these parameters can be evaluated against known physiological thresholds.

#### 2.2.2. Influence of the Nozzle Diameter on the Volume of a Droplet

**Figure 4:**
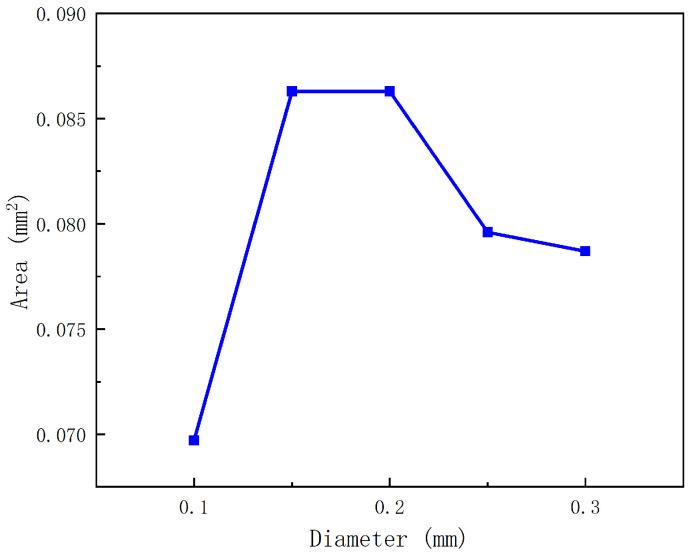
Influence of nozzle diameter on droplet formation.

The nozzle diameter is a significant parameter affecting the droplet volume. Interestingly, the droplet volume does not increase linearly with increasing nozzle diameter. As shown in Figure 4, the droplet area (used here as a 2D proxy of droplet volume in the axisymmetric CFD model) increases as the nozzle diameter increases from 0.10 mm to 0.15 mm, remains approximately unchanged between 0.15 mm and 0.20 mm, and then decreases when the nozzle diameter further increases from 0.20 mm to 0.30 mm. For the smaller nozzles (0.10–0.20 mm), increasing the nozzle diameter enlarges the flow cross-sectional area, which can facilitate liquid delivery during each actuation cycle and thus increase (or maintain) the ejected droplet size within this range. When the nozzle diameter exceeds 0.20 mm (i.e., 0.25–0.30 mm in Figure 4), the characteristic shear rate in the nozzle is expected to decrease; given the shear-thinning behavior of the bioink (Section 2.1), the apparent viscosity correspondingly increases, which may reduce the effectiveness of jetting and lead to a smaller droplet. Because the CFD analysis is based on a two-dimensional model, droplet volumes are represented by droplet areas in the solution volume-fraction contours.

#### 2.2.3. Influence of the Stroke Length on the Volume of a Droplet

**Figure 5:**
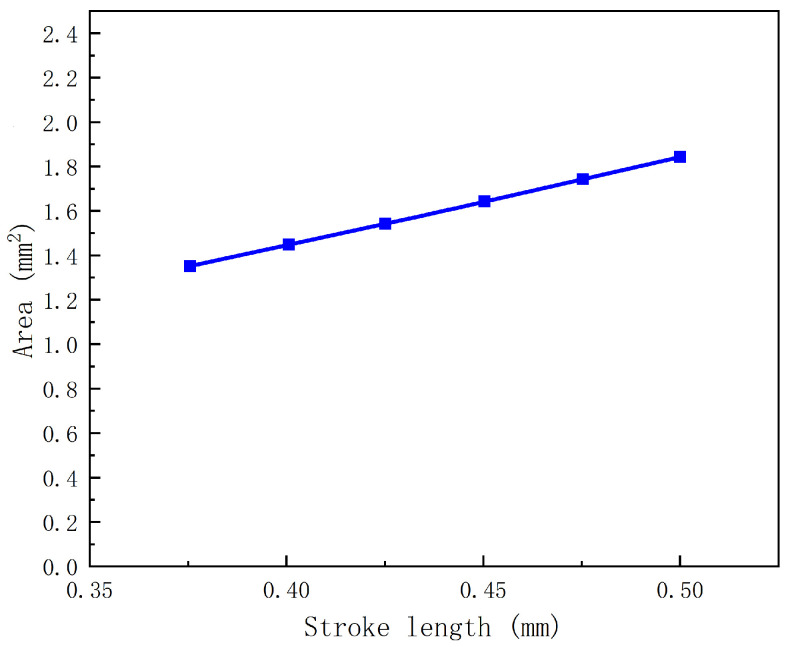
Influence of stroke length on droplet volume.

The stroke length is a key parameter that strongly determines the droplet volume. In general, a longer stroke length presses more solution out of the nozzle. Figure 5 shows the droplet volume under varying stroke lengths, and the results indicate that the droplet volume increases approximately linearly with increasing stroke length. Because the CFD analysis is based on a two-dimensional axisymmetric model, droplet volumes are represented by droplet areas extracted from the solution volume-fraction contours. In the proposed dispenser, the stroke length defines the maximum displacement of the mobile anchor and thereby the opening extent of the microvalve during each actuation cycle. Under the same actuation timing and inlet back pressure adopted in the CFD model, a larger stroke length corresponds to a larger valve opening, which can increase the amount of liquid delivered through the nozzle per cycle and thus produces a larger droplet. Within the tested stroke-length range where stable droplet formation is maintained, the increase in delivered liquid per cycle scales approximately with stroke length, leading to the near-linear trend observed in Figure 5. We note that this linearity is an approximation within the investigated window, and deviations may occur outside the stable jetting range.

#### 2.2.4. Influence of the Velocity of the Mobile Anchor on the Volume of a Droplet

**Figure 6:**
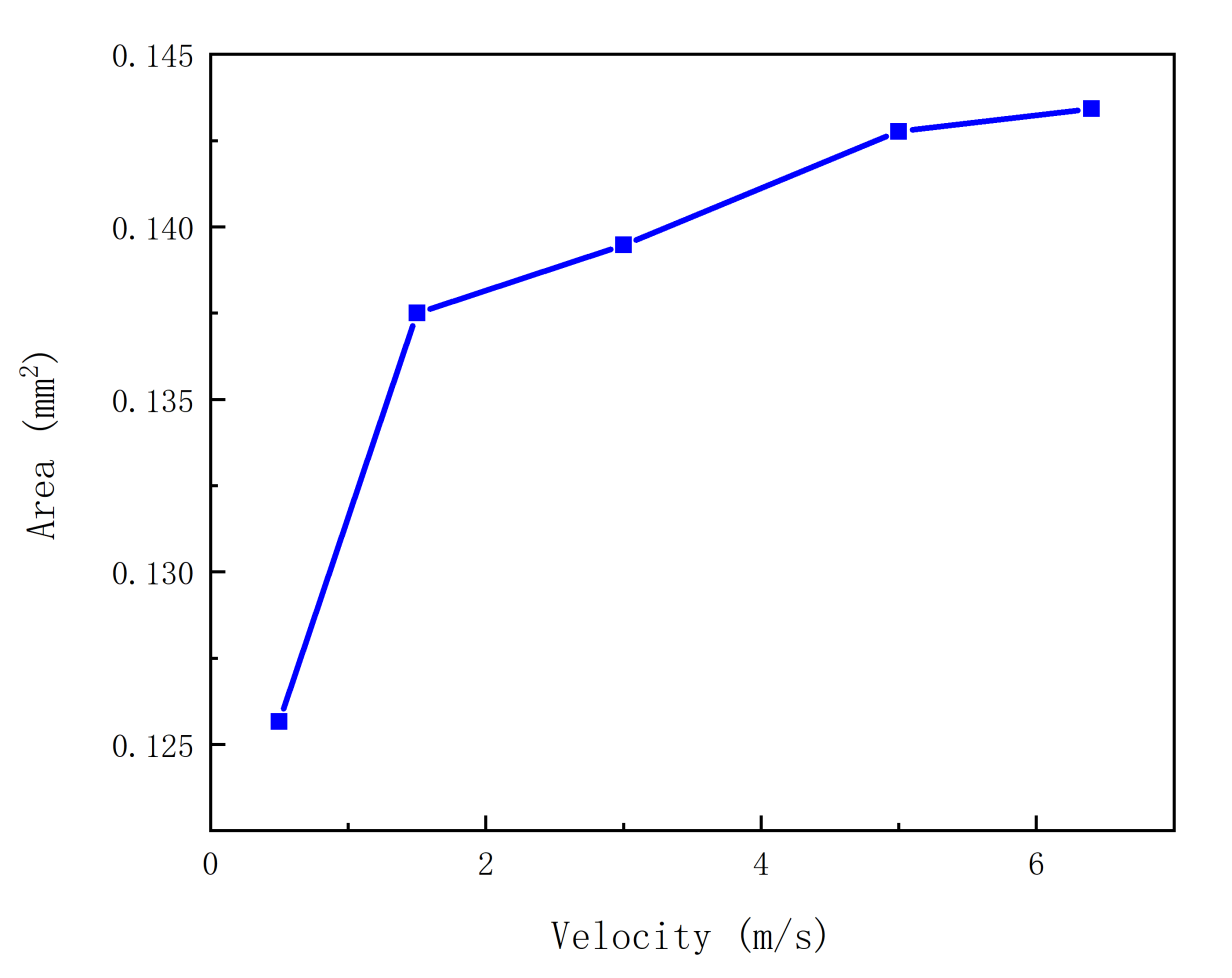
Influence of the mobile-anchor velocity on droplet volume.

The velocity of the mobile anchor is another key design parameter. In general, a higher mobile-anchor velocity produces a higher flow velocity of the solution, which facilitates droplet formation. Figure 6 compares the droplet volume at different mobile-anchor velocities. When the mobile-anchor velocity is very low, stable droplet ejection is difficult to achieve. As the velocity increases, the droplet volume increases gradually.

#### 2.2.5. Influence of the Flow Passage Length on the Volume of a Droplet

**Figure 7:**
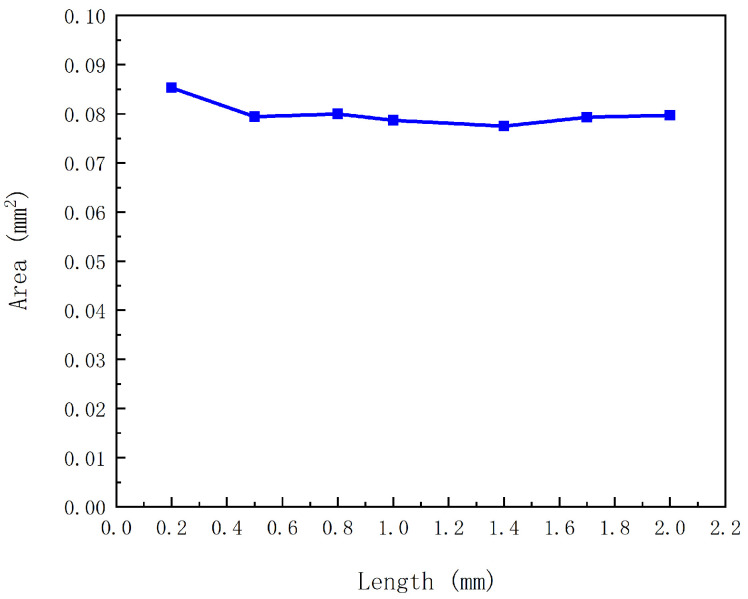
Influence of the flow-passage length on droplet volume.

Figure 7 shows that changes in the flow-passage length have nearly no influence on droplet volume (represented by droplet area in the axisymmetric CFD results). This weak sensitivity can be understood based on the CFD configuration and boundary conditions used in this study.The computational domain is restricted to the local region surrounding the mobile-anchor tip and the nozzle, enabling detailed examination of the flow and necking behavior near the nozzle exit, while a fixed back pressure is applied at the inlet. Under the same actuation cycle and fixed inlet back pressure, varying the upstream flow-passage length within the tested range does not significantly modify the transient flow response in the near-nozzle region; consequently, the ejected volume per cycle and the resulting droplet area remain almost unchanged. This also indicates, from another perspective, that the nozzle diameter and stroke length are the decisive factors governing droplet volume when other design parameters remain constant.

### 2.3. Dynamic Modeling and Analysis of the Electromagnetic Valve

#### 2.3.1. Influence of the Stroke Length on the Velocity of Mobile Anchor

**Figure 8:**
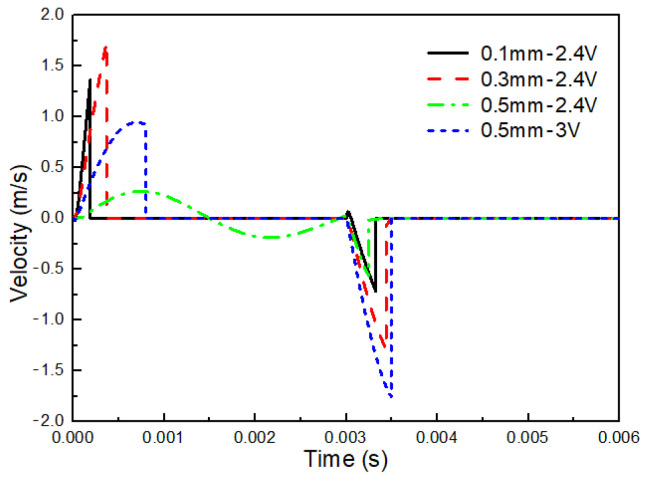
Velocityprofiles of the mobile anchor with varying stroke lengths.

The stroke length of the mobile anchor is an important design parameter that has a significant influence on its motion. Figure 8 compares the velocity profiles of the mobile anchor for different stroke lengths. Except for the parameters indicated in the legend, all other parameters are identical to those listed in Table 1. As shown in Figure 8, the velocity curves exhibit distinct characteristics with varying stroke lengths. By comparing the cases with stroke lengths of 0.1 mm and 0.3 mm, it can be observed that the mobile-anchor velocity increases more sharply for the smaller stroke length, resulting in a shorter acceleration time before the mobile anchor reaches the stationary anchor. This behavior arises because the electromagnetic force acting on the mobile anchor is strongly dependent on its distance from the stationary anchor; a shorter distance produces a higher electromagnetic force. Consequently, the acceleration of the mobile anchor is higher when the stroke length is smaller.

When the stroke length is further increased to 0.5 mm, the mobile-anchor velocity varies in a sinusoidal manner over the time interval of 0–0.003 s, and no abrupt velocity drop is observed. This indicates that the electromagnetic force is insufficient to overcome the spring force due to the increased distance between the stationary anchor and the mobile anchor, preventing contact between them. This issue can be addressed by increasing the coil voltage, as a higher voltage significantly enhances the electromagnetic field intensity. Figure 8 also presents the velocity profile for a stroke length of 0.5 mm at a coil voltage of 3 V, where the velocity drops abruptly to 0 m/s at approximately 0.0007 s. These simulation results suggest that the stroke length of the mobile anchor should not be designed to be excessively large unless a higher coil voltage is applied to generate sufficient electromagnetic force. However, higher coil voltages can lead to increased heat generation in the coil. As this study focuses on the jetting process of cell-free bioinks, the specific implications for cell viability are not assessed here but warrant consideration in future biological applications.

#### 2.3.2. Influence of the Preload of Spring on the Velocity of Mobile Anchor

**Figure 9:**
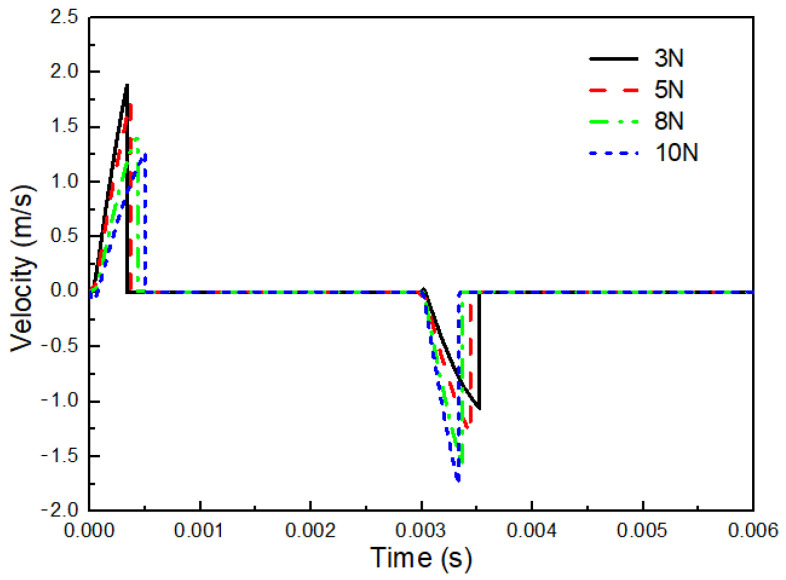
Velocity profiles of the mobile anchor with varying spring preload.

The spring preload acting on the mobile anchor is another important parameter that strongly influences its motion. Figure 9 compares the velocity profiles of the mobile anchor under different spring preload values. As shown, both the acceleration and peak velocity of the mobile anchor decrease gradually with increasing spring preload during its motion toward the stationary anchor. This indicates that the microvalve exhibits a faster response during the opening process when a lower spring preload is applied. In contrast, when the mobile anchor moves toward the valve seat, increasing the spring preload enhances both the acceleration and the peak velocity, implying that the microvalve responds more rapidly during the closing process with a higher spring preload.

The above behavior can be explained as follows. During the microvalve opening process, the resultant force acting on the mobile anchor is larger when a lower spring preload is used, assuming all other parameters remain unchanged, thereby accelerating the mobile anchor more rapidly. Similarly, during the microvalve closing process, a higher spring preload produces a larger resultant force on the mobile anchor, leading to greater acceleration. These results indicate that the overall dynamic response of the microvalve during both opening and closing processes should be considered when selecting an appropriate spring preload.

#### 2.3.3. Influence of the Spring Stiffness on the Velocity of Mobile Anchor

**Figure 10:**
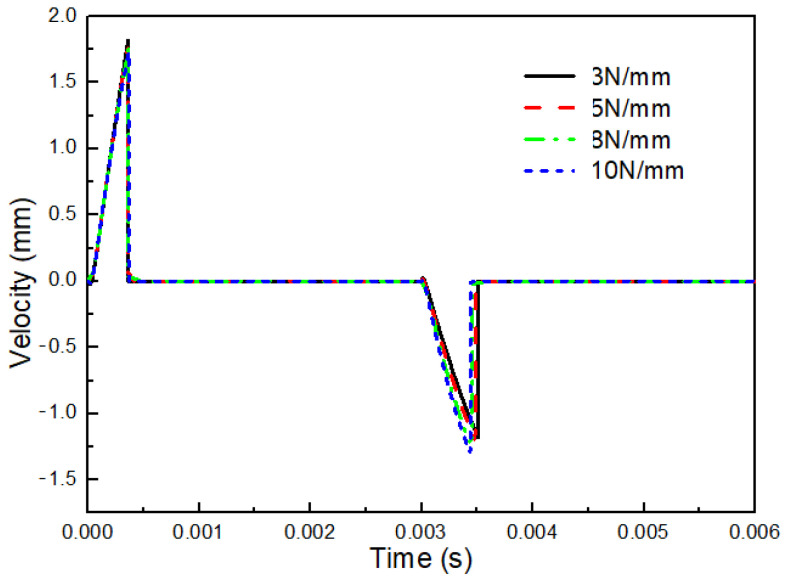
Velocity profiles of the mobile anchor with varying spring stiffness.

The spring stiffness is also an important parameter in determining the initial spring length and its initial precompression. Figure 10 presents the velocity profiles of the mobile anchor for different spring stiffness values, indicating that spring stiffness has little influence on the mobile-anchor velocity within the range of 3–10 N/mm. As shown, the velocity profiles during the opening process are almost identical for all cases. In addition, during the closing process, both the acceleration and peak velocity of the mobile anchor increase only slightly with increasing spring stiffness. Therefore, enhancing the spring stiffness is not an effective approach to improving the rapid response performance of the microvalve.

### 2.4. Electromagnetic Field Analysis of the Electromagnetic Valve

#### 2.4.1. FEM Results of the Electromagnetic Field of Dispenser

**Figure 11:**
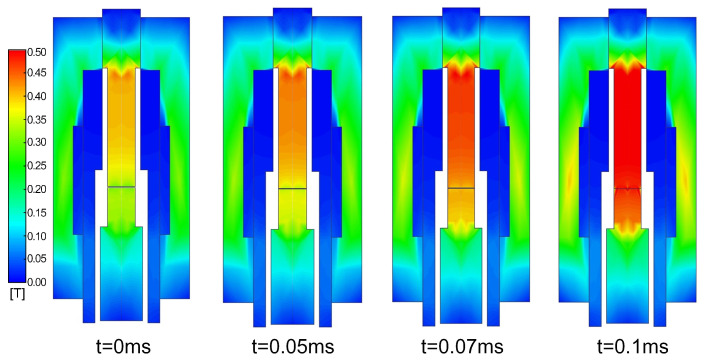
Time-varying electromagnetic field of the dispenser.

Ansys Maxwell (Ansys Electronics Desktop, Release 2024 R1; Ansys, Inc., Canonsburg, PA, USA) is employed to solve the governing equations of the electromagnetic field. Figure 11 presents the time-varying electromagnetic field at different moments within the time interval of 0–0.1 ms. At t=0 ms, the initial air gap between the stationary anchor and the mobile anchor is 0.3 mm. When the coil is energized, the magnetic field distribution is non-uniform, and the magnetic field strength is significantly enhanced in regions composed of soft magnetic materials. As the mobile anchor moves toward the stationary anchor under the electromagnetic force, the air gap between them decreases. Because the air gap strongly affects the magnetic field distribution, a pronounced increase in magnetic field strength at both the stationary and mobile anchors is observed as the air gap narrows.

#### 2.4.2. Influence of the Height of Coil L on the Electromagnetic Force of Coil

**Figure 12:**
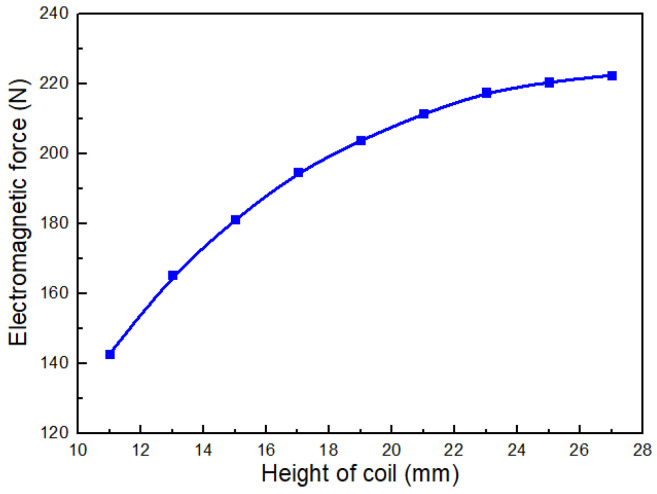
Electromagnetic force of the coil for varying coil heights *L*.

The coil height *L* is an important design parameter that directly determines the structural dimensions of the dispenser. Provided that sufficient electromagnetic force can be generated to actuate the mobile anchor, the coil height should be minimized to reduce the overall size of the dispenser. Figure 12 shows the electromagnetic force acting on the mobile anchor for different coil heights *L*. As shown, the electromagnetic force increases rapidly at first and then more gradually as *L* increases within the range of 11 mm to 27 mm. This behavior can be understood from the magnetic-flux-driven nature of the actuation: increasing *L* increases the effective flux linkage in the magnetic circuit and reduces the overall reluctance of the energized region, thereby increasing the magnetic flux Φ and the electromagnetic force. According to Equation (2), $F_{e}$ increases with Φ, so a pronounced force gain is observed when *L* is small. When *L* becomes larger, the incremental contribution of additional coil height diminishes, which may be attributed to magnetic saturation in the soft-magnetic components and increased flux leakage, leading to a gradually flattening trend in Figure 12. When L=11mm, the electromagnetic force reaches approximately 140 N, which is sufficient to overcome the spring preload under the modeled operating conditions.

### 2.5. Influence of the Ampere-Turn of Coil on the Electromagnetic Force of Coil

**Figure 13:**
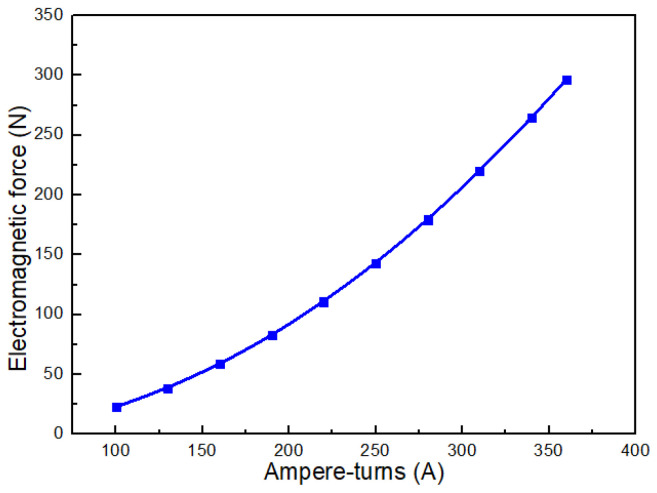
Electromagnetic force of the coil with varying ampere-turns.

The ampere-turns of the coil is a key parameter that determines the coil’s capability to generate a magnetic field. It is defined as the product of the number of coil turns and the electric current flowing through the coil. In general, a larger ampere-turns value results in a stronger magnetic field. Figure 13 illustrates the electromagnetic force generated by the coil for different ampere-turns values. The results indicate that the electromagnetic force increases rapidly as the ampere-turns of the coil increase. These findings provide guidance for the appropriate design of the coil ampere-turns.

#### Influence of the Radius of Mobile Anchor on the Electromagnetic Force of Coil

**Figure 14:**
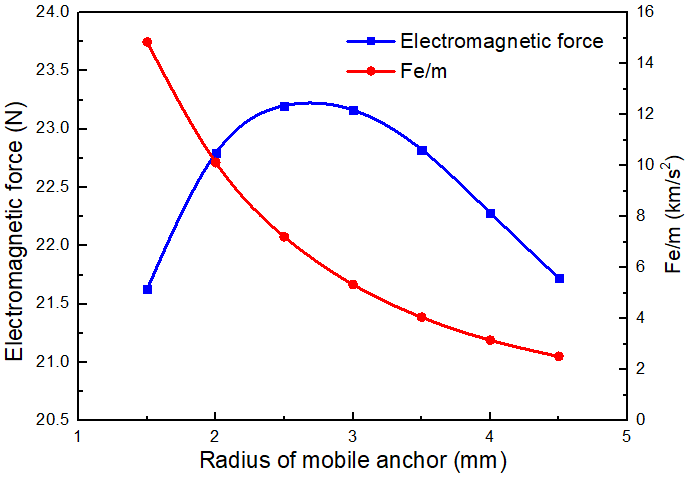
Electromagnetic force and $F_{e}/m$ as functions of the mobile-anchor diameter.

The diameter of the mobile anchor not only significantly affects its mass but also strongly influences the magnetic field of the dispenser. As shown in Figure 14, the electromagnetic force first increases and then decreases as the radius of the mobile anchor increases. The electromagnetic force reaches its maximum when the radius of the mobile anchor is 2.5 mm. However, the mass of the mobile anchor also increases with increasing radius, implying that the acceleration of the mobile anchor may exhibit a different trend.

To further evaluate the acceleration capability of the mobile anchor, the ratio of the electromagnetic force $F_{e}$ to the mass of the mobile anchor *m* is calculated, as also shown in Figure 14. Although the electromagnetic force initially increases and then decreases with increasing radius, the ratio $F_{e}/m$ decreases monotonically due to the increasing mass. These results suggest that the radius of the mobile anchor should be minimized to achieve higher acceleration.

### 2.6. Printing Experiment with the Proposed Dispenser Prototype

#### 2.6.1. The Influence of Various Stroke Lengths on the Volume of Droplets

**Figure 15:**
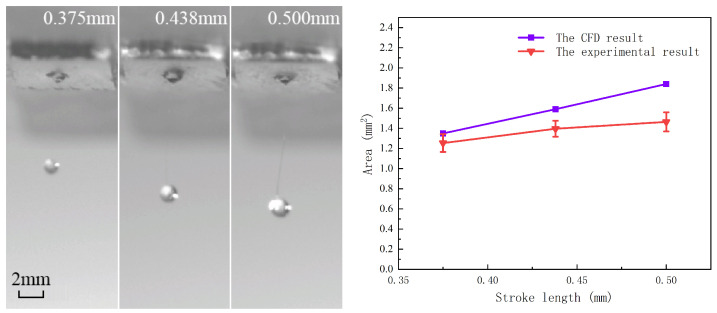
The influence of the stroke length. (Each experimental result was determined by averaging the projected area of ten droplets generated in a continuous and stable ejection sequence.)

A 2.5% sodium alginate solution was used to conduct the printing experiments. According to the CFD analysis, the droplet size increases with increasing stroke length. Therefore, three stroke lengths (0.375 mm, 0.438 mm, and 0.5 mm) were selected as experimental parameters. A subsequent CFD analysis was then performed using these experimental settings. Figure 15 presents photographs of droplets produced at different stroke lengths along with the corresponding CFD results. Because solution loss during experiments is inevitable, the CFD predictions and experimental measurements do not match perfectly. Nevertheless, the results consistently confirm that the droplet volume increases with increasing stroke length.

#### 2.6.2. The Printing Trials of Little Tubes

During the printing process, the sodium alginate solution was extruded and deposited in droplets using the dispenser prototype. After printing, the sodium alginate structures were cross-linked using a calcium chloride (CaCl_2_) solution to solidify the printed shapes. The printed structures were immersed in a 2% (*w*/*v*) CaCl_2_ solution for 1 hour at room temperature (25 °C), allowing for complete crosslinking. The crosslinking time was selected based on preliminary tests that showed it provided optimal structural stability and material properties.

To prevent unwanted spreading of the material during the printing process, the extrusion pressure was carefully controlled. Additionally, the freshly printed structures were placed in a chilled PBS buffer solution immediately after printing to limit expansion and maintain the desired printed shapes prior to crosslinking.

**Figure 16:**
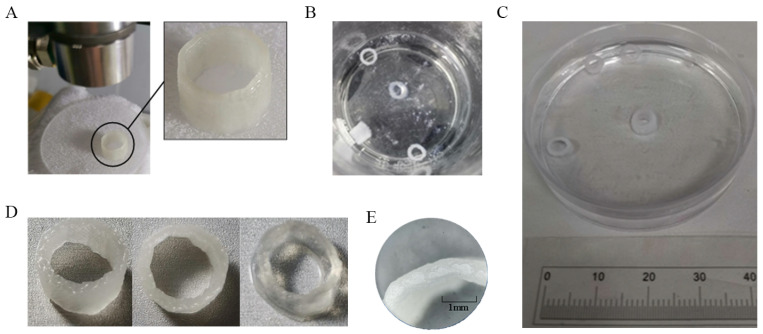
Printing trials of small tubes. (**A**) Freshly printed model; (**B**) Frozen model in a chilled 10% PBS ice–water mixture; (**C**) Cross-linked model after incubation in PBS at 37 °C for 1 h; (**D**) Cross-linked model showing melting/deformation without storage in 1% PBS buffer; (**E**) Dimensional characterization: designed outer diameter 5 mm; measured average outer diameter (n = 3) 5.68 mm (13.56% deviation); wall thickness 0.40 mm.

The printing trials were carried out using a three-axis Cartesian robotic stage. First, 10% PBS buffer was placed in a refrigerator for 30 min until it formed an ice–water mixture. Second, the freshly printed model (Figure 16A,E) was placed in the chilled PBS mixture, and the PBS containing the model was refrigerated for an additional 20 min. Third, the frozen model was cross-linked in PBS buffer in an incubator at 37 °C for 1 h (Figure 16B,C). Finally, the model was removed and stored in a 1% PBS buffer solution to maintain its shape. As shown in Figure 16D, the cross-linked model melted easily without the protection of the 1% PBS buffer solution.

For the printed tube structures in Figure 16B,C, the outer diameter was designed to be 5 mm. The average outer diameter measured from three printed tubes was 5.68 mm, which corresponds to an error rate of approximately 13.56%. The wall thickness of the tube was 0.40 mm (Figure 16E).

## 3. Conclusions

In this study, a transient electromagnetic-valve-actuated jetting strategy was developed to overcome the intrinsic incompatibility between high-viscosity shear-thinning bioinks and the limited printable viscosity window of drop-on-demand inkjet systems. By integrating axisymmetric transient CFD, valve dynamics modeling, electromagnetic finite-element analysis, and prototype experiments using a 2.5% (*w*/*v*) sodium alginate solution, the following conclusions can be drawn:

1. **Droplet-formation mechanism and key sensitivities.** The CFD results elucidated the stage-wise evolution of droplet formation (pressurization/ligament formation, necking, and pinch-off) and quantified the influence of key parameters. Within the investigated stable jetting regime, the droplet volume (represented by droplet area in the axisymmetric model) exhibits a non-monotonic dependence on nozzle diameter (Figure 4): it increases from 0.10 mm to 0.15 mm, remains approximately unchanged between 0.15 mm and 0.20 mm, and then decreases as the nozzle diameter further increases from 0.25 mm to 0.30 mm. Meanwhile, droplet volume increases approximately linearly with stroke length (Figure 5). In contrast, varying the flow-passage length in the tested range shows negligible influence on droplet volume (Figure 7).
2. **Actuation dynamics of the anchor–spring system.** The dynamic model captures the rapid transient motion of the mobile anchor under electromagnetic excitation and spring restoring force. For the baseline configuration, the anchor reaches a peak opening velocity of approximately 1.7 m/s and a peak closing velocity of approximately 1.4 m/s, supporting the transient, valve-actuated nature of the proposed jetting strategy and providing guidance for selecting stroke length and spring parameters.
3. **Electromagnetic design implications.** Electromagnetic simulations show that increasing coil height enhances the electromagnetic force with diminishing returns. Notably, at L=11mm, the electromagnetic force is approximately 140 N, which is sufficient to overcome the spring preload ($F_{0}=5\;N$) under the modeled conditions. These results provide quantitative guidance for coil-parameter design.
4. **Prototype validation and printing demonstrations.** Based on the above design guidance, a dispenser prototype was built with a representative parameter set (e.g., microvalve stroke length 0.3 mm, spring preload 5 N, coil height 11 mm, and coil ampere-turns 100 A). A predefined current waveform enabled the microvalve to be opened and closed within 0.5 ms, supporting rapid response while reducing coil heating. Experiments using 2.5% sodium alginate confirmed stable droplet generation and tunable droplet size via stroke length (tested at 0.375 mm, 0.438 mm, and 0.5 mm), and demonstrated the fabrication of three-dimensional tubular structures.

Overall, this work provides a coupled ‘material–flow–actuation’ framework and practical design guidelines for droplet-based printing of high-viscosity shear-thinning hydrogel inks, focusing on process design and characterization. Importantly, while this study may provide an engineering basis for future bioprinting applications, the objectives of the present work are confined to the manufacturing/jetting-process development and physicochemical/printing characterization of the (cell-free) hydrogel ink. However, it is important to clarify that this study does not involve cell-laden printing or any biological validation (e.g., cell viability assessment). The primary focus of this work is on the droplet generation/jetting process of high-viscosity shear-thinning hydrogel inks. Biological evaluation (including cell-related studies such as cell viability) is beyond the scope of the present manuscript and will be investigated in future work.

## 4. Materials and Methods

### 4.1. The Working Principle of an Electromagnetic-Valve-Actuated Dispenser

**Figure 17:**
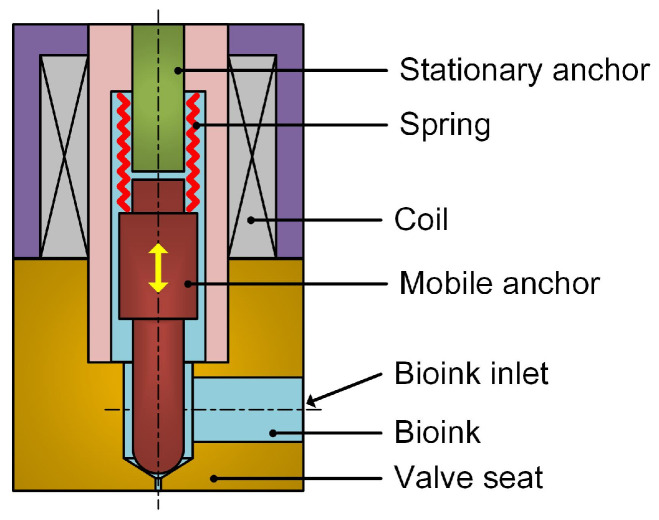
Schematic diagram of an electromagnetic-valve-actuated dispenser.

Figure 17 demonstrates the schematic diagram of the proposed dispenser. It mainly consists of a stationary anchor, a spring, a coil, a mobile anchor, and a valve seat. Under working conditions, the mobile anchor is electromagnetically actuated by the coil to produce reciprocating motion with high-frequency and a short stroke, and the bioink is ejected from the dispenser when the mobile anchor moves toward the valve seat. The detailed process is described as follows. At the initial state, the coil is powered off, the mobile anchor is in sealing contact with the valve seat under the action of the spring, and the microvalve remains closed. When the coil is powered on, the mobile anchor compresses the spring, moves toward the stationary anchor, and finally remains in contact with the stationary anchor under the electromagnetic force. Meanwhile, the biomaterials are fed into the nozzle inlet by pressurized air. The coil is then powered off again, and the electromagnetic force disappears. The mobile anchor moves toward the valve seat under the action of the compressed spring. A local high-pressure region forms near the nozzle inlet due to the impact of the mobile anchor; the biomaterials around the nozzle are accelerated and ultimately ejected from the nozzle as a droplet. This process repeats continuously, and the bioink is ejected drop-by-drop from the nozzle.

The feasibility of the proposed dispenser highly depends on the high-frequency motion of the mobile anchor, which requires collaborative design of the dynamic performance of its moving parts, electromagnetic design of coil, hardware design of circuit and proper electric control. In the following section, the flow simulation, dynamics analysis and electromagnetic analysis are implemented to guide the design of some crucial parameters of dispenser, such as the valve stroke length and the stiffness of spring.

### 4.2. CFD Modeling for the Printing Process

**Figure 18:**
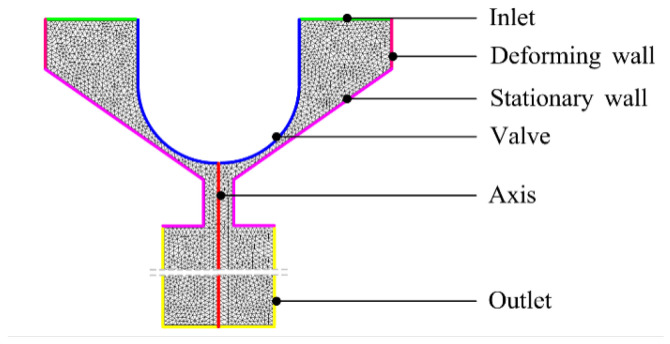
CFD model for the electromagnetic-valve-driven inkjet system.

To investigate how key structural parameters (e.g., nozzle diameter and length) affect single-droplet formation, this study develops a two-dimensional axisymmetric transient CFD model to simulate multiple actuation cycles of an electromagnetic-valve-driven inkjet system, as shown in Figure 18.

The computational domain is restricted to the small region surrounding the spherical tip of the mobile anchor, the nozzle, and a localized external area. This design allows detailed examination of the flow field and necking behavior near the nozzle exit. Local mesh refinement and small time steps are used around the nozzle interface, and a back pressure of 6500 Pa is applied at the inlet. Initially, the liquid fully occupies the nozzle channel, and the mobile anchor presses the valve cone against the seat, sealing the nozzle. During each actuation cycle, the anchor first retracts from the valve seat and reaches its maximum displacement at 1 ms, opening the nozzle. It then remains stationary for 2 ms before moving back toward the valve seat over the next 2 ms, gradually closing the nozzle.

### 4.3. Dynamics Modeling of the Electromagnetic Valve

**Figure 19:**
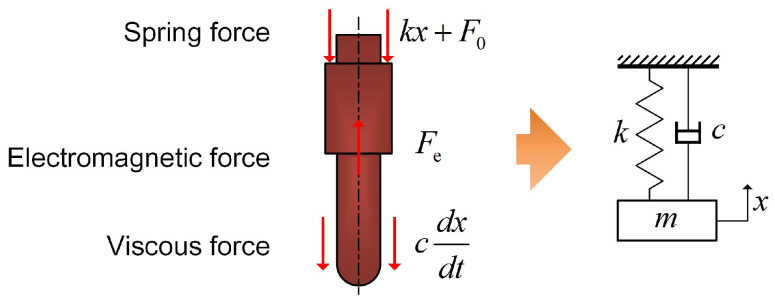
Free-body diagram of the mobile anchor and its simplified dynamic model.

Figure 19 illustrates the free-body diagram of the mobile anchor under working conditions. Several forces act on the anchor, including the spring preload $F_{0}$, the spring restoring force kx induced by the anchor displacement, the electromagnetic force $F_{e}$, and the viscous force from the bioink. The mobile anchor has a very small mass and a millimeter-level stroke; therefore, its weight has a negligible effect on its vibration characteristics and is neglected here. Because the mobile anchor vibrates only in one direction, the system can be simplified as a single-degree-of-freedom oscillator. Accordingly, the equation of motion of the mobile anchor can be written as Equation (1).

Where *m* represents the mass of the mobile anchor, *k* is the stiffness of the spring, and *c* is the viscous force coefficient. ζ is a coefficient indicating the working state of the coil: ζ=1 when the coil is powered on; otherwise, ζ=0.

(1) $$m\frac{d^{2}x}{dt^{2}}=\zeta F_{e}-kx-F_{0}-c\frac{dx}{dt}$$

The electromagnetic force $F_{e}$ can be calculated based on Equation (4), where Φ represents the magnetic flux, $\mu_{0}$ is the permeability of vacuum, and *A* represents the area of the end face of the mobile anchor.

(2) $$F_{e}=\frac{\Phi^{2}}{2\mu_{0}A}$$

(3) $$U\left(t\right)=I\left(t\right)\;\cdot \;R+N\frac{d\Phi }{dt}$$

(4) $$NI\left(t\right)=\frac{\Phi \left(x_{\max}-x\right)}{\mu_{0}A}$$

The initial and boundary conditions for the equation of motion of the mobile anchor can be written as Equation (5).

(5) $$\frac{dx}{dt}=\left\{\begin{array}{cc} 0, & t=0,\\ 0, & x=0,\\ 0, & x=x_{\max}. \end{array}\right.$$

**Figure 20:**
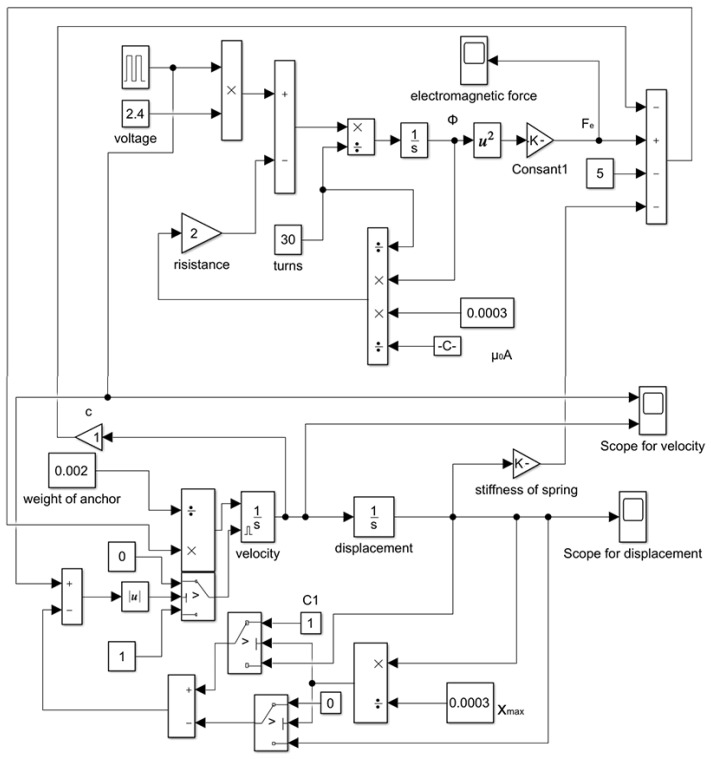
Simulinkmodel.

To solve the equation of motion of the mobile anchor, the Simulink module of MATLAB R2024b (The MathWorks, Inc., Natick, MA, USA) was employed to simulate the anchor motion. The simulation model is shown in Figure 20. Key initial parameters are listed in Table 1. To improve clarity, it is noted that the parameter values in Table 1 are selected as the nominal (baseline) configuration used in our simulations and are informed by the prototype design (e.g., geometric dimensions) and practical driving constraints. In particular, the anchor end-face area *A* is determined by the mobile-anchor radius in the prototype design, and the stroke limit $x_{\max}$ corresponds to the designed valve stroke. The anchor mass *m* is taken from the fabricated/dimensioned moving part, reflecting the millimeter-scale lightweight anchor assumed in the dynamic model. The spring preload $F_{0}$ and stiffness *k* are chosen according to the spring setting that ensures reliable sealing at the initial state and provides sufficient restoring force during valve closure. The electrical parameters *R* (coil resistance), *N* (coil turns), and *U* (driving voltage) are taken from the coil/circuit configuration adopted in our actuation experiments. These nominal values are used as the baseline in Section 2.3.1, Section 2.3.2 and Section 2.3.3, where one parameter is varied at a time while the others are fixed at the Table 1 values.

**Figure 21:**
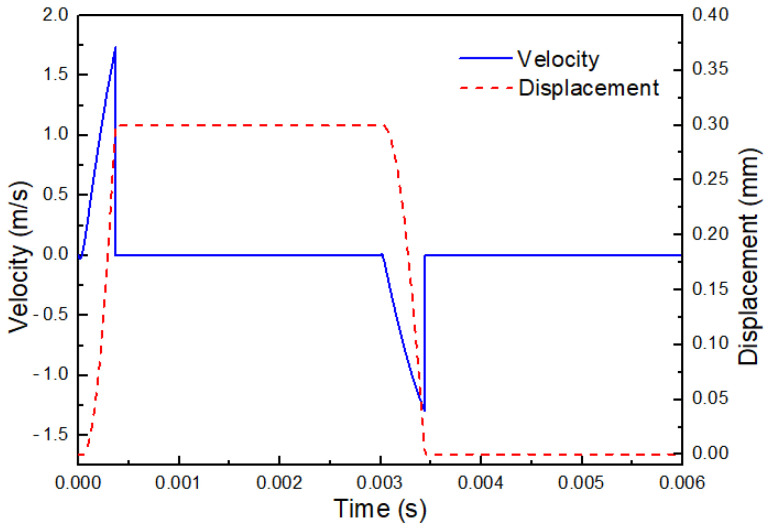
Motion of the mobile anchor in one actuation cycle.

Figure 21 illustrates the motion of the mobile anchor over one actuation cycle obtained from the previously established simulation model. As shown, the velocity of the mobile anchor increases sharply under the action of the electromagnetic force, as expected. The velocity reaches a maximum value of 1.7 m/s and then drops rapidly to 0 m/s once the mobile anchor comes into contact with the stationary anchor. Subsequently, the mobile anchor remains at zero velocity under the sustained electromagnetic force, and its position stays fixed at the upper end of the stroke, thereby maintaining the microvalve in an open state.

After 0.003 s, the mobile anchor moves toward the valve seat under the action of the spring force due to the sudden disappearance of the electromagnetic force. Its velocity increases in the opposite direction and reaches a maximum value of 1.4 m/s before contacting the valve seat. The mobile anchor is then stopped by the valve seat and remains at the lower end of the stroke for a short duration, maintaining the microvalve in a closed state.

### 4.4. FEM Modeling of the Electromagnetic Field of Dispenser

Several crucial design parameters, including the coil height *L*, the ampere-turns of the coil NI, the number of coil turns *N*, and the diameter of the mobile anchor *r*, have significant effects on the electromagnetic field of the dispenser and the dynamic performance of the microvalve. In addition, the materials of individual components also strongly influence the electromagnetic field of the dispenser. These effects cannot be accurately captured using theoretical equations alone. Therefore, in this section, the electromagnetic field of the dispenser is investigated using the finite element method.

**Figure 22:**
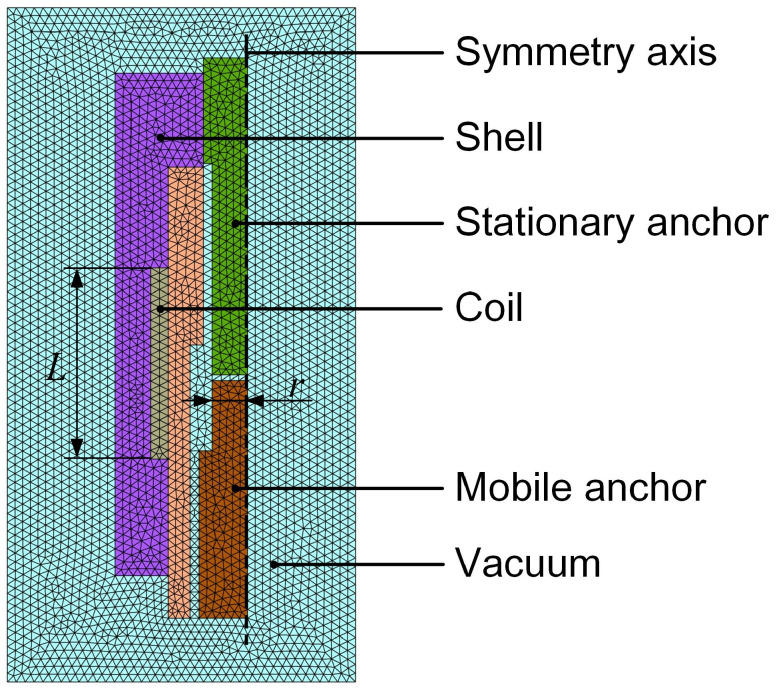
Finite element model of the dispenser.

Figure 22 illustrates the finite element model employed to simulate the electromagnetic field of the dispenser. Owing to the cyclic symmetry of the dispenser, a two-dimensional planar model is adopted to reduce computational cost. Iron is selected for the stationary anchor, mobile anchor, and shell to enhance the magnetic field generated by the electric current, as it is a soft magnetic material that can be easily magnetized and demagnetized. The coil is made of copper, while the remaining components are fabricated from stainless steel.

### 4.5. A Prototype of the Proposed Dispenser

#### 4.5.1. Configuration of Dispenser Prototype

**Figure 23:**
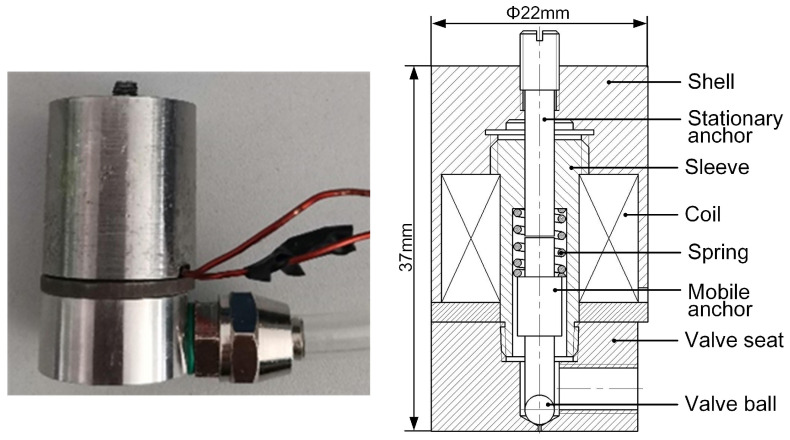
Prototype of the dispenser.

According to the above analysis, a prototype of an electromagnetic-valve-actuated dispenser was built, and its detailed configuration is shown in Figure 23. The soft magnetic material DT4C was used to fabricate the shell, stationary anchor, and mobile anchor, while stainless steel 304 was used to manufacture the spring, sleeve, and valve seat. Because the hardness of DT4C is much lower than that of stainless steel 304, a valve ball was inlaid at the lower end of the mobile anchor to prevent damage during repeated impacts. The valve ball was made of ruby to provide higher hardness. The sleeve is connected to the valve seat and shell via threaded joints. The stationary anchor has a clearance fit with the sleeve and is connected to the shell using a fine thread with a pitch of 0.5 mm. Therefore, the valve stroke length can be adjusted by changing the axial position of the stationary anchor.

The key design parameters of the dispenser are summarized as follows: the microvalve stroke length is 0.3 mm, the spring preload is 5 N, the spring stiffness is 5 N/mm, the coil height is 11 mm, the coil ampere-turns is 100 A, and the radius of the mobile anchor is 1.5 mm.

#### 4.5.2. Circuit Hardware Design and Electric Control Design

**Figure 24:**
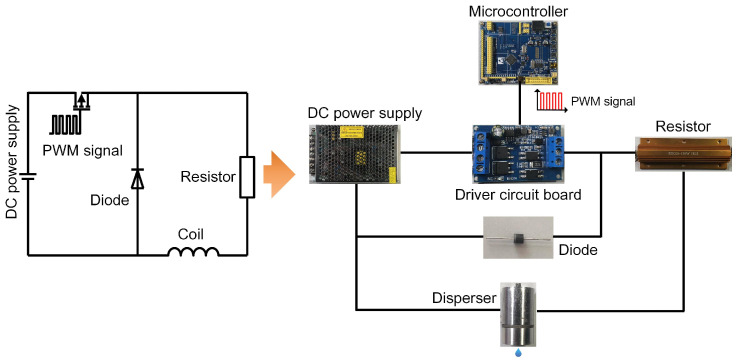
Hardware design of the driving circuit.

To supply an appropriate current to the coil of the dispenser, the circuit hardware is designed as shown in Figure 24. The system consists of a direct current (DC) power supply, a microcontroller, a driver circuit board, a resistor, a diode, and the coil of the dispenser. Under operating conditions, the microcontroller generates a pulse-width modulation (PWM) signal for the driver circuit board. By adjusting the frequency and duty cycle of the PWM signal, the output current of the driver circuit board can be regulated to meet the design requirements. It should be noted that when the coil current is abruptly interrupted, the induced electromotive force generates a reverse voltage that may damage circuit components. To prevent this, a diode is incorporated into the circuit to protect the electronic elements.

**Figure 25:**
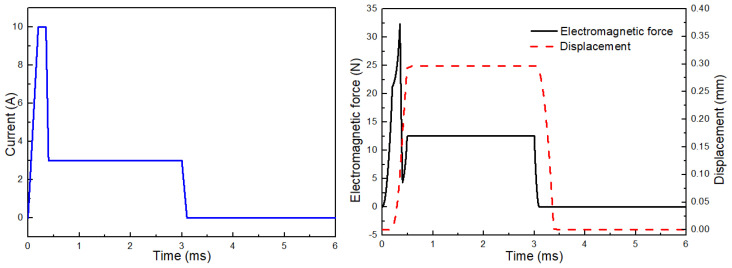
Electric control design.

To achieve a fast response of the microvalve while maintaining a low coil heat-generation rate, a predefined current waveform is adopted in this study, as shown in Figure 25. According to the above analysis, the mobile anchor can be accelerated rapidly by applying a high current during the microvalve opening stage. Once the microvalve is open, it is unnecessary to maintain a large holding current; instead, a lower current is sufficient to keep the mobile anchor in position, which helps reduce the coil heat-generation rate. The electromagnetic force and displacement profiles of the mobile anchor are also presented in Figure 25. As shown, the electromagnetic force increases sharply and then decreases rapidly during the opening process, after which it remains approximately constant to hold the mobile anchor. The displacement curve indicates that the microvalve can be opened and closed within 0.5 ms, demonstrating excellent dynamic response. Therefore, by using a predefined current waveform, both rapid microvalve response and reduced coil heating can be achieved. It is noted that while this current waveform strategy aims to reduce average coil heating, direct temperature measurement of the nozzle area during prolonged operation is not within the scope of this study, which focuses on the jetting process characterization of cell-free bioinks.

## Figures and Tables

**Table 1:** Initial parameters of the dispenser.

Parameter	Value
*A*	$7.06\;{\mathrm{mm}}^{2}$
*m*	2g
$x_{\max}$	0.3mm
$F_{0}$	5N
*k*	10N/mm
*R*	0.2Ω
*N*	30
*U*	2.4V

## Data Availability

The original contributions presented in this study are included in the article. Further inquiries can be directed to the corresponding authors.
